# Supplementary material for: Physiological and Molecular Characteristics of Southern Leaf Blight Resistance in Sweet Corn Inbred Lines
Source: Int J Mol Sci. 2022 Sep 6;23(18):10236. doi: 10.3390/ijms231810236 (PMC9499663; doi:10.3390/ijms231810236)
Supplement: Supplementary file 1 [file ijms-23-10236-s001.zip › Supplementary Figures.pdf]

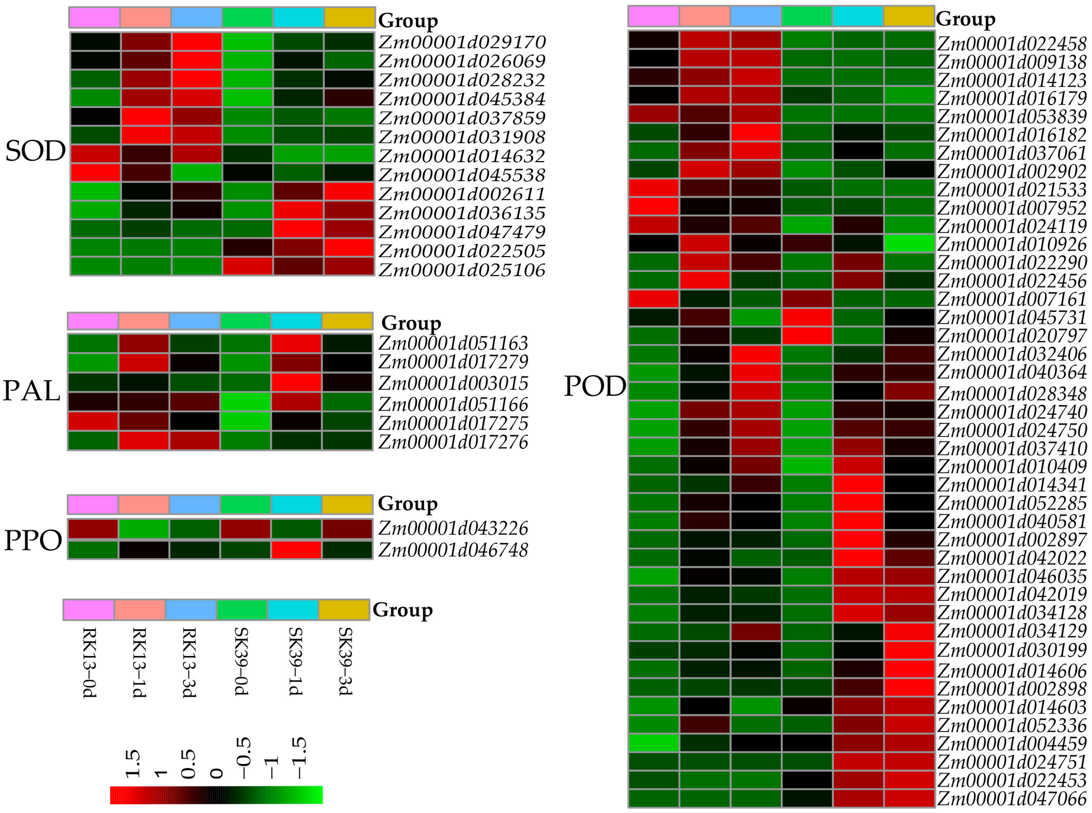

Figure S1. Gene expression patterns of protective enzymes.

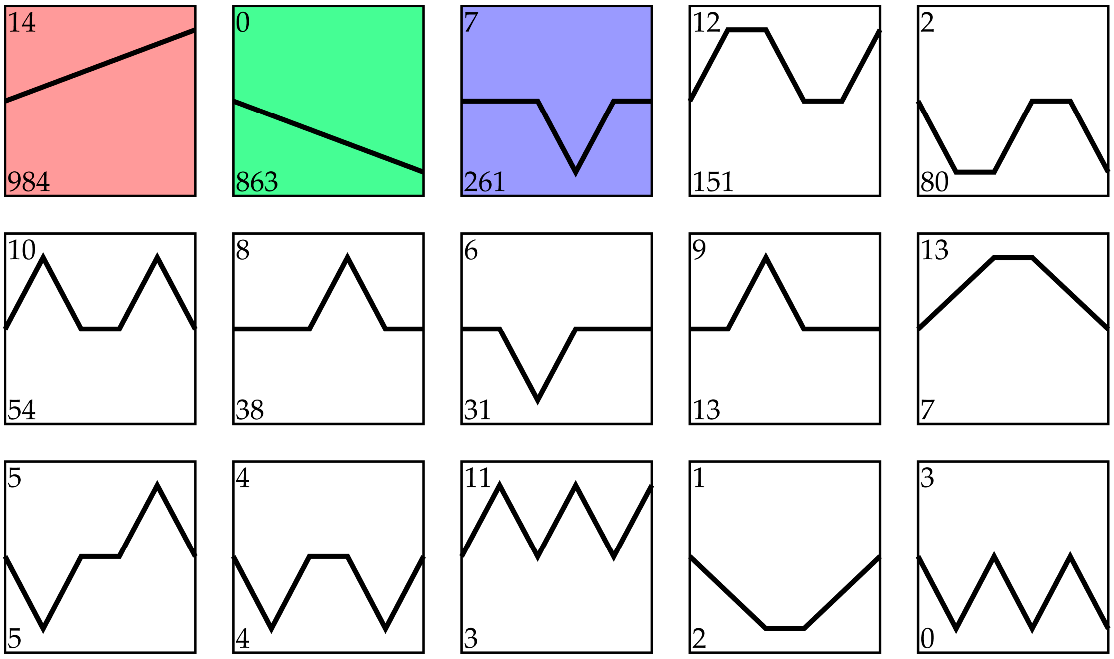

Figure S2. Gene expression patterns across time points (RK13-0d/1d/3d, SK39-0d/1d/3d). Colored ones represented significant trends. The number in the upper left of each trend represented the trend number, and the number in the lower left represented the number of genes that fit the trend. The black line represented the expression trend.
